# Essential roles of autophagy in metabolic regulation in endosperm development during rice seed maturation

**DOI:** 10.1038/s41598-019-54361-1

**Published:** 2019-12-06

**Authors:** Yuri Sera, Shigeru Hanamata, Shingo Sakamoto, Seijiro Ono, Kentaro Kaneko, Yuudai Mitsui, Tomoko Koyano, Naoko Fujita, Ai Sasou, Takehiro Masumura, Hikaru Saji, Ken-Ichi Nonomura, Nobutaka Mitsuda, Toshiaki Mitsui, Takamitsu Kurusu, Kazuyuki Kuchitsu

**Affiliations:** 1 Department of Applied Biological Science, Tokyo University of Science, 2641 Yamazaki, Noda, Chiba, 278-8510 Japan; 2 Graduate School of Science and Technology, Niigata University, 2-8050 Ikarashi, Niigata, 950-2181 Japan; 3 Bioproduction Research Institute, National Institute of Advanced Industrial Science and Technology (AIST), Tsukuba, Ibaraki, 305-8566 Japan; 4 Plant Cytogenetics Laboratory, National Institute of Genetics, 1111 Yata, Mishima, Shizuoka, 411-8540 Japan; 5 Department of Biological Production, Akita Prefectural University, 241-438 Shimoshinjo Nakano Kaidobata-Nishi, Akita City, Akita, 010-0195 Japan; 6 Laboratory of Genetic Engineering, Graduate School of Life and Environmental Sciences, Kyoto Prefectural University, Shimogamo, Kyoto, 606-8522 Japan; 7 Center for Environmental Biology and Ecosystem Studies, National Institute for Environmental Studies (NIES), Onogawa, Tsukuba, Ibaraki, 305-8506 Japan; 8 Department of Mechanical and Electrical Engineering, Suwa University of Science, 5000-1 Toyohira, Chino, Nagano, 391-0292 Japan; 9 Imaging Frontier Center, Tokyo University of Science, 2641 Yamazaki, Noda, Chiba, 278-8510 Japan; 10 Present Address: Division of Mucosal Immunology, Institute of Medical Science, The University of Tokyo, 4-6-1 Shirokanedai, Minato-ku, Tokyo, 108-8639 Japan; 11 Present Address: Department of Developmental Biology, Institute for Plant Sciences and Microbiology, University of Hamburg, 22609 Hamburg, Germany

**Keywords:** Seed development, Abiotic

## Abstract

Autophagy plays crucial roles in the recycling of metabolites, and is involved in many developmental processes. Rice mutants defective in autophagy are male sterile due to immature pollens, indicating its critical role in pollen development. However, physiological roles of autophagy during seed maturation had remained unknown. We here found that seeds of the rice autophagy-deficient mutant *Osatg7-1*, that produces seeds at a very low frequency in paddy fields, are smaller and show chalky appearance and lower starch content in the endosperm at the mature stage under normal growth condition. We comprehensively analyzed the effects of disruption of autophagy on biochemical properties, proteome and seed quality, and found an abnormal activation of starch degradation pathways including accumulation of *α*-amylases in the endosperm during seed maturation in *Osatg7-1*. These results indicate critical involvement of autophagy in metabolic regulation in the endosperm of rice, and provide insights into novel autophagy-mediated regulation of starch metabolism during seed maturation.

## Introduction

Rice (*Oryza sativa* L.) is one of the most important crops in the world not only in agriculture but as a model monocot in scientific research. Elucidating the molecular mechanism for the development of its seed/starch endosperm is of particular significance for food production. Endosperm development in rice consists of four stages: coenocytic nuclear division, cellularization, differentiation of an outer aleurone layer and an inner starchy endosperm, and accumulation of starch, proteins and lipids *etc*.^1–6^. Starchy endosperm cells occupy most of the space in the caryopsis and provide the main energy source of rice seeds^4,6,7^. Starch granules are tightly packed in the endosperm to form translucent grains. However, rice grains exposed to excess environmental stresses during ripening as well as flowering stages exhibit a whitish chalky appearance, which causes serious damage to grain quality^8^ and economical loss because they impair the commercial value^9^.

Autophagy is an intracellular destructive mechanism evolutionarily conserved among eukaryotes, degrading intracellular proteins, metabolites, and organelles for recycling and quality control^10^. Isolation membranes emerge in the cytosol to develop a double membrane structure called the autophagosomes to enclose intracellular components, which fuses with the lytic compartments such as the vacuole and lysosomes^11^. Many of the more than 30 autophagy-related genes (*ATG*) identified in yeast, are conserved in most eukaryotes, including animals and plants^12^.

Autophagy is essential in growth, development, and cell survival in many eukaryotes^13,14^. In plants, autophagy has been suggested to play roles in the recycling of proteins and metabolites, including lipids, at the whole-plant level, and is involved in many physiological processes such as plant nutrition, senescence, hormonal responses, pathogen resistance, stress protection, seed germination, photomorphogenesis, chloroplast maturation, tracheary element formation, and fertile floret development under nutrient-limited conditions^15–20^. Moreover, autophagy has recently been shown to be induced in the tapetum in anthers at the uninucleate stage and is required for postmeiotic anther development including the programmed cell death (PCD)-mediated degradation of the tapetum and pollen maturation in rice^21,22^. However, physiological roles of autophagy during seed maturation and its relationship with metabolic regulation in endosperm development had remained poorly understood in plants.

We herein discovered that autophagy deficiency in rice leads to chalky appearance of grains even under normal growth condition. Multidisciplinary analyses including microscopic, biochemical, genetic and proteomic analyses revealed that this is due to misregulation of sugar and starch metabolism mediated at least in part by abnormal upregulation of *α*-amylase during seed maturation. These results indicate critical roles of autophagy in seed maturation, grain quality control during reproductive development in rice, and suggest the impact of controlling autophagy in agriculture.

## Results and Discussion

### Chalky appearance of grains of the *Osatg7-1* mutant under normal growth conditions

**Figure 1:**
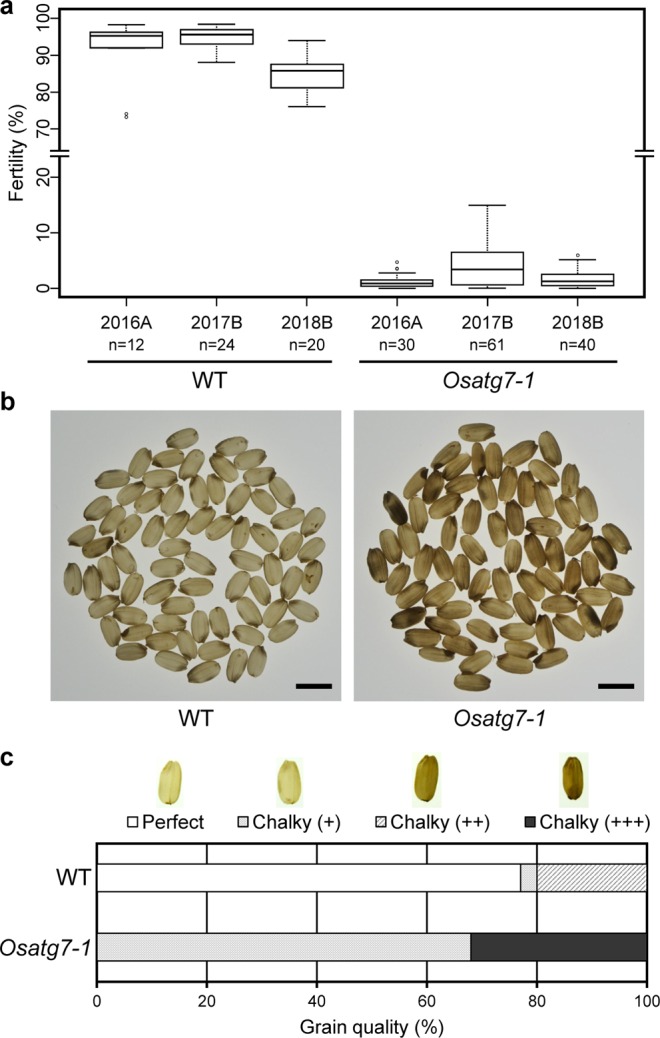
Effects of autophagy disruption on rice reproductive development grown in the paddy field. (**a**) The *Osatg7-1* mutant exhibited a severe sterile phenotype under both greenhouse (2016A) and paddy field conditions (2017B and 2018B). The Box limits represent the upper and lower quartiles, the bold line represents the median, and the whiskers the minimum and maximum within 1.5 IQR of the lower or the upper quartile values. The unfilled circles indicate to suspected outliers. *n* = 12–61 independent samples. (**b**) The *Osatg7-1* mutant had grains with a chalky appearance. Photographs were taken under back lighting. Scale bar: 5 mm. (**c**) Viable chalky grains shown in (**b**) were quantified and categorized. Data are the mean of 100 seeds of rice.

Rice autophagy-deficient mutants (*Osatg7-1, Osatg7-2* and *Osatg9*) show severe male sterility, fail to accumulate lipidic and starch components in pollen grains at the flowering stage, exhibit reduced pollen germination activity, and have limited anther dehiscence, indicating that autophagy is involved in pollen maturation in rice^21–23^. The *Osatg7-1* mutant showed complete sporophytic male sterility in regular growth chambers and greenhouses or under pot cultivation outdoors^21^. On the other hand, we have found that the *Osatg7-1* mutant produce seeds at a very low frequency in a specific greenhouse (Fig. 1a, 2016A) or paddy fields (Fig. 1a, 2017B and 2018B). The fertility rate of the *Osatg7-1* mutant (1.3 ± 1.3 {n = 30}, 2016A; 4.3 ± 4.1{n = 61}, 2017B; 1.8 ± 1.7{n = 40}, 2018B) was much lower than those of the wild type (WT; 91.6 ± 8.6{n = 12}, 2016A; 94.8 ± 2.9 {n = 24}, 2017B; 84.7 ± 4.7 {n = 20}, 2018B).

We investigated these seeds in order to understand if autophagy plays any roles in seed developmental processes in rice, and found that the majority of the *Osatg7-1* had grains with whitish chalky appearance under normal growth condition in the paddy field (Fig. 1b). Under this condition (2017B), 77% of WT seeds were perfect grains, while 0% in *Osatg7-1* and all grains showed chalky appearance (Fig. 1c). The *Osatg7-1* grains were smaller than WT, and the weight of grains of the *Osatg7-1* mutant (20.10 ± 2.09^**^ [mg]; n = 120, ^**^P value < 0.01) were slightly smaller than those of WT grains (20.79 ± 1.05 [mg]; n = 120). These phenotypes of the autophagy-deficient mutant were quite similar to those observed in the WT seeds suffering from heat stress during seed maturation^24^. These results indicate that autophagy plays critical roles in seed development and maturation in rice.

### Abnormal morphology of starch granules in the endosperm of the *Osatg7-1* mutant

**Figure 2:**
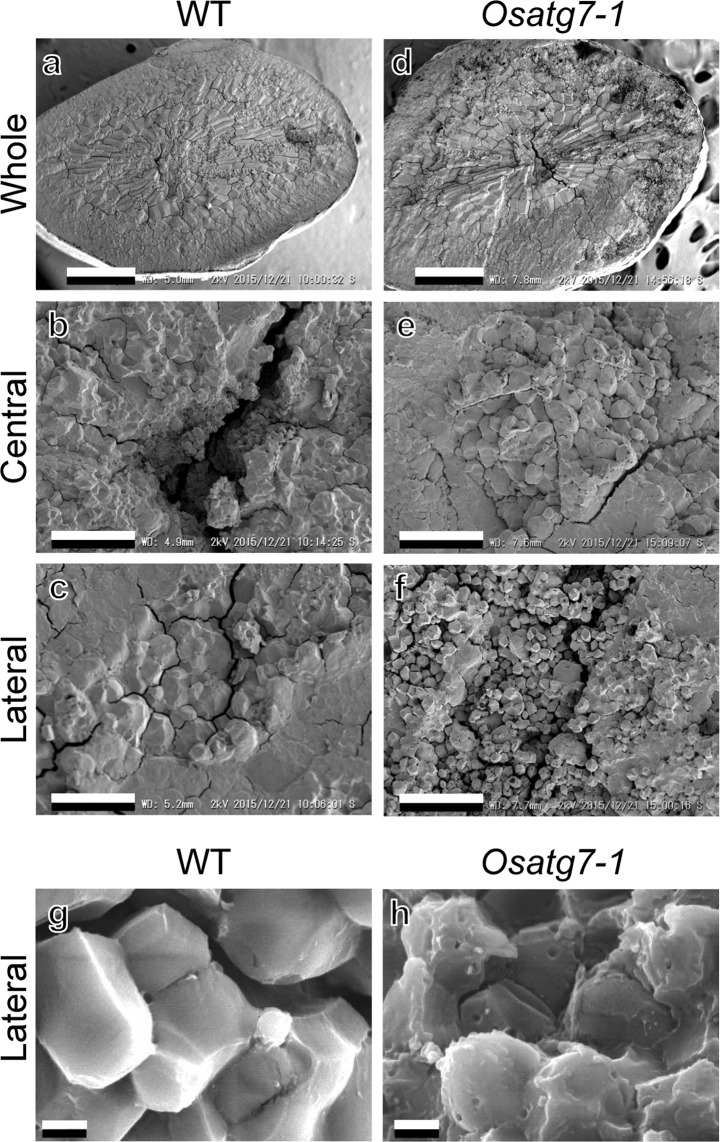
Abnormal morphology of starch granules in the endosperm of the *Osatg7-1* mutant. (**a–f**) The ultrastructure of mature seeds in WT (left) and the *Osatg7-1* mutant (right). Close up of central (**b**,**e**) and lateral (**c**,**f**) regions of whole images (**a,d**). Images were taken by SEM. Scale bars: 500 μm (**a**,**d**) and 30 μm (**b**,**c**,**e**,**f**). Starch granules at the lateral region of the endosperm were smaller and sparser in the *Osatg7-1* mutant than in WT. (**g–h**) Close up of starch granules at the lateral region. Images were taken by EPMA. Scale bars: 2 μm. The starch granules of the *Osatg7-1* mutant had small pits. Similar results were obtained from 5 independent samples.

Chalkiness is shown to be derived from the opaque portion in the rice endosperm due to the loose packing of starch granules and protein bodies^25,26^. We performed a scanning electron microscopic (SEM) analysis to reveal the starch granule morphology of the *Osatg7-1* mutant. As shown in Fig. 2, WT starch granules were tightly packed in the whole endosperm, and their shape was polygonal with sharp edges (Fig. 2a–c). On the contrary, in the chalky grains of the *Osatg7-1* mutant, the starch granules of the central region had a similar shape and tight packing to that of WT (Fig. 2e), whereas those of the mutant in the lateral part were loosely packed and several small pits were often observed on the surface of the starch granules (Fig. 2f). These small pits of the mutant were also confirmed by electron probe micro analysis (EPMA) (Fig. 2h). The size of starch granules in the lateral region of the endosperm of WT and *Osatg7-1* were 4.67 ± 1.33 µm and 2.09 ± 0.53 µm, respectively, indicating that starch granules in the lateral region of the endosperm were smaller and sparser in the *Osatg7-1* mutant than in WT. These phenotypes of the autophagy-deficient mutant appear to share some similarities with those of the wild type seeds suffering from heat stress during ripening^24^ (Fig. 2f).

A transmission electron microscopic (TEM) analysis was performed to elucidate the ultrastructure of protein bodies (PBs) in the endosperm of mature rice grains. In the mature endosperm, subcellular structures (PB-I, PB-II, and starch granules) in sectioned rice grains were perfectly preserved in WT and the *Osatg7-1* mutant. PB-I was observed in the endosperm as large, spherical structures with diameters of 1–3 μm, whereas PB-II was electron-dense, irregularly-shaped structures with diameters of 2–4 μm (Supplementary Fig. 1). Therefore, no significant differences were noted in the size or morphology of PBs in the starch endosperm between the *Osatg7-1* mutant and WT (Supplementary Fig. 1). These results indicate that the main cause of the chalky grains of the *Osatg7-1* mutant is the abnormal (small and sparse) morphology of starch granules.

### Involvement of autophagy in the regulation of starch and sugar metabolism during rice seed maturation

**Figure 3:**
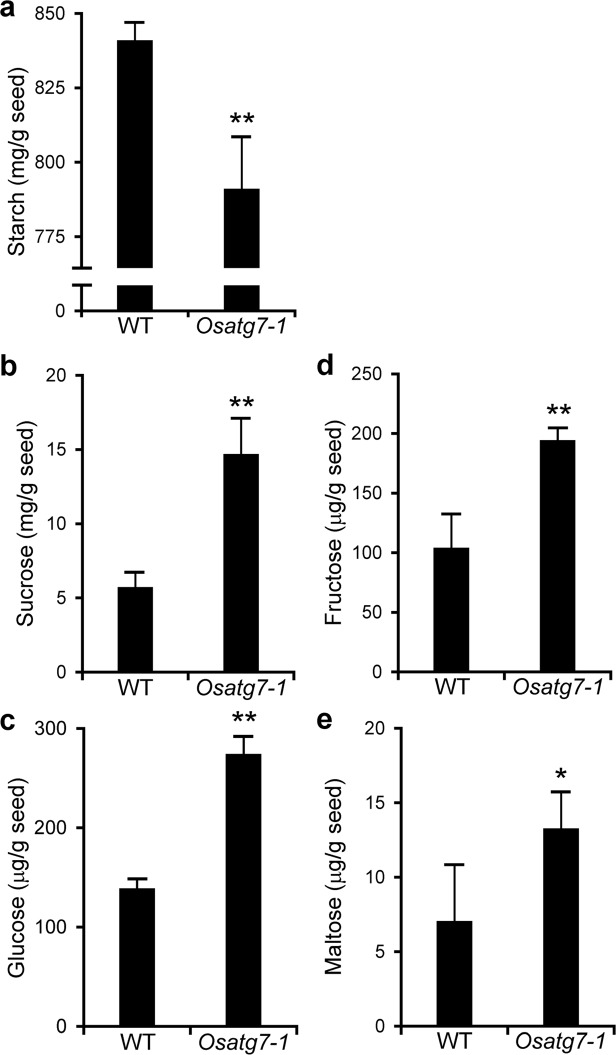
Effects of autophagy disruption on starch and sugar contents in mature grains of rice. Compositions of starch and soluble sugar contents in the grains of WT and the *Osatg7-1* mutant at the mature stage. The amounts of starch (**a**), sucrose (**b**), glucose (**c**), fructose (**d**), and maltose (**e**) were measured. Data are means ± SD; *n* = 4 independent samples. *P < 0.05, **P < 0.01; significantly different from the controls.

Quality of rice is essentially influenced by the chemical composition of the storage of starch and protein derived from nitrogen (N) and carbon (C) metabolism during grain filling^27^. Autophagy contributes to efficient N and C remobilization at the whole-plant level and plays an important role in the biomass production of rice^28^. To investigate the effects of disruption of autophagy on the sugar and starch contents of mature seeds, we conducted comprehensive analyses on the soluble sugar and starch levels of grains at the mature stage in the *Osatg7-1* mutant and WT. Endogenous level of starch in grains were lower in the *Osatg7-1* mutant than in WT (Fig. 3a), while the levels of a number of soluble sugars were higher in *Osatg7-1* (Fig. 3b–e). Maltose, a typical degradation product of starch in the endosperm, also accumulated in the chalky grains of the *Osatg7-1* mutant (Fig. 3e), suggesting that the abnormal activation of starch degradation occurs in the endosperm of the autophagy-deficient mutant, *Osatg7-1*.

**Figure 4:**
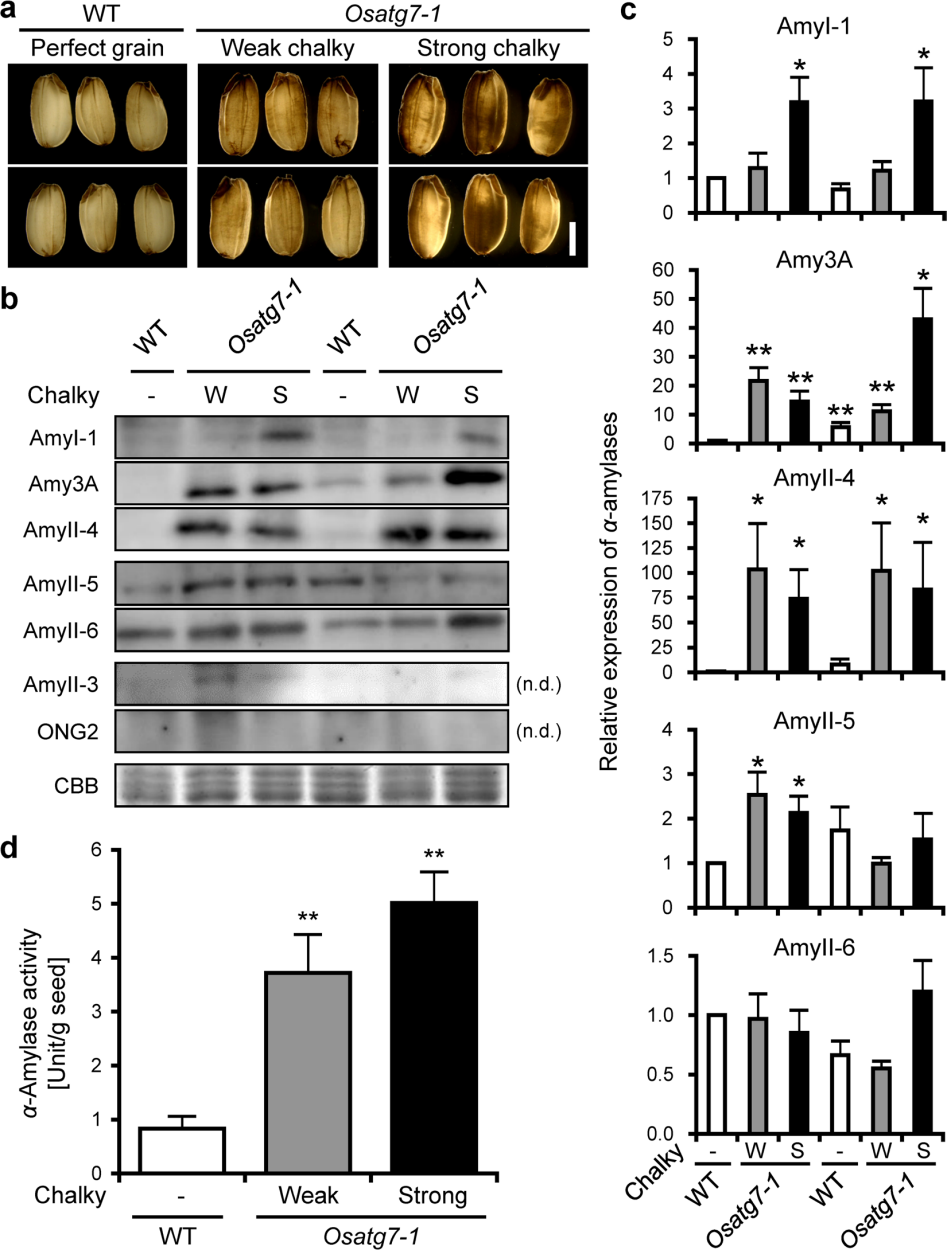
Effects of autophagy disruption on the expression and activity of *α*-amylases in mature grains of rice. (**a**) A photograph of the chalky grains of the *Osatg7-1* mutant at a mature stage grown in the paddy field. Pictures were taken under backlighting. (**b**) Immunoblotting images. Whole chalky grains at a mature stage were subjected to protein analysis, followed by SDS-PAGE and immunoblotting with specific antibodies^28^. Left and right parts indicate the sample of 2016 and 2017, respectively. The perfect proteins of Amy-II-3 and ONG2 were not detected (n.d.). CBB, Coomassie Brilliant Blue. W and S indicate a weak and strong chalky grain phenotype, respectively. The full-length blots are presented in Supplementary Figures 2 and 3. (**c**) Quantitation of *α*-amylase expression. The amount of each *α*-amylase isoform in WT grains was normalized to 1. Data are means ± SD; *n* = 4 independent samples. *P < 0.05; significantly different from the controls. (**d**) Quantitation of *α*-amylase activity in WT and *Osatg7-1* mutant grains at a mature stage. Enzyme activity was corrected by the protein content of the respective extracts. Data are means ± SD; *n* = 4 independent samples. **P < 0.01; significantly different from the controls.

Rice has at least eight members of *α*-amylase, which are classified into three subfamilies: Amy1, Amy2 and Amy3 and play a role in the first committed step for the degradation of reserve starch^29,30^. We then characterized the amounts of *α*-amylase isoforms in the chalky grains of the *Osatg7-1* mutant at a mature stage by immunoblot analysis (Fig. 4a–c). The results obtained showed that AmyI-1 (Amy1A) and AmyII-4 (Amy3D) were more strongly expressed in the chalky grains of the *Osatg7-1* mutant than in WT (Fig. 4b,c). Moreover, the expression of *α*-amylase 3 A, which is one of the enzymes potentially involved in this chalky appearance through the degradation of starch accumulating in the developing grain, was also enhanced in *Osatg7-1* grains, while no significant difference was observed for the other amylases (AmyII-3, II-5, and II-6) or *α*-glucosidase (ONG2) between WT and the *Osatg7-1* mutant (Fig. 4b,c). As expected, the enzymatic activity of *α*-amylases was also higher in *Osatg7-1* grains than in WT at a mature stage (Fig. 4d). These data were consistent with a previous study showing that the ectopic overexpression of *α*-amylases, such as *Amy1A* and *Amy3D*, resulted in chalky grains even under normal growth conditions^31^. Furthermore, Amy1A and Amy3D were shown to be mainly localized in the outer layers of rice grains, while *α*-glucosidase and AmyII-3 were mainly localized in the inner layers^24^. Since the *Osatg7-1* mutant has starch granules with an abnormal morphology at the lateral region of the endosperm, the abnormal expression and activation of *α*-amylases may occur at the lateral region of the *Osatg7-1* endosperm during rice seed maturation.

### Effects of autophagy disruption on starch quality in the endosperm during rice seed maturation

Starch quality is an important parameter influencing the quality of rice grains. Previous studies indicated that environmental stresses, including temperature, at the grain filling stage alter the starch composition of rice grains^32^. Furthermore, the contents and fine structures of amylose and amylopectin in starch were shown to affect the physicochemical characteristics and textural properties of rice grains^33^. Therefore, we characterized the starch composition of the chalky grains of the *Osatg7-1* mutant using the gel-filtration chromatography^34^. The results obtained showed no significant differences in the contents and structure of amylose and amylopectin in starchy endosperms between the *Osatg7-1* mutant and WT grains (Supplementary Table 1), suggesting that the reasons for the loosely-packed starch granules of the *Osatg7-1* mutant were factors other than the chain-length distribution of amylopectin.

### Effects of autophagy disruption on the proteome of the endosperm in rice grains

**Figure 5:**
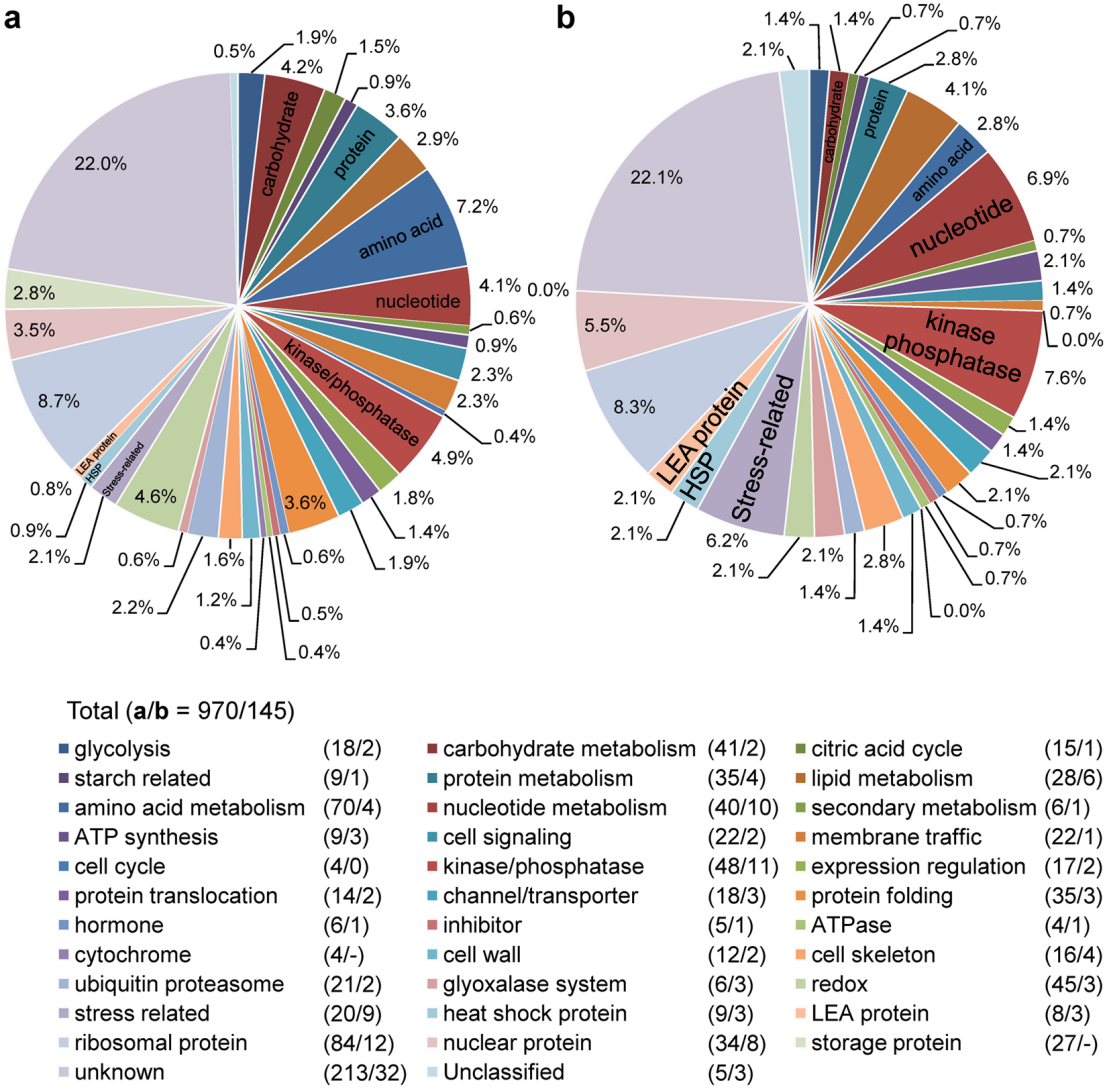
Effects of autophagy disruption on the proteome of the mature grains of rice. Classification of proteins whose expression differed by more than 1.5-fold in mature grains between WT and the *Osatg7-1* mutant. Whole seeds were subjected to protein extraction, followed by shotgun proteomic analysis with iTRAQ labeling. Proteins were categorized using UniProtKB database search. (**a**/**b**): (**a**) Total identified proteins (970); (**b**) >1.5-fold up- or down-regulated proteins in the *Osatg7-1* mutant grains (145).

We performed a quantitative shotgun proteomic analysis of starchy endosperms prepared from *Osatg7-1* chalky grains and WT grains. The proteins extracted were trypsin-digested and labeled using iTRAQ (isobaric tag for relative and absolute quantitation), followed by a tandem mass spectrometry (MS/MS) analysis^24^. We identified 970 proteins extracted from *Osatg7-1* and WT grains. Among them, 145 proteins, 14.9% of all identified proteins, were deregulated (more than a 1.5-fold difference from WT normal grains; *P* value < 0.05) in *Osatg7-1* chalky grains (Fig. 5 and Supplementary Table 2), suggesting possible involvement of autophagy in the expression of these proteins. Among this population, 130 proteins were up-regulated and 15 were down-regulated (Supplementary Table 2).

To investigate the biological processes affected by the disruption of autophagy in rice, an analysis of proteins using the InterProScan search was performed. The annotation of protein sequences using InterProScan permitted them to fall into 33 categories including energy and metabolic pathways and the molecular functional group (Fig. 5 and Supplemental Table 2). Our results showed that autophagy affects the carbohydrate, protein, amino acid and nucleotide metabolism. Since autophagy is an intracellular destructive mechanism that degrades intracellular proteins and metabolites for recycling, the source of amino acids may be depleted in the chalky grains of *Osatg7-1* mutant. In the molecular functional group, stress-related proteins, HSPs (heat shock proteins) and LEA (late embryogenesis abundant) proteins were ranked at the top of the category, suggesting that the *Osatg7-1* mutant gets stressed even under normal growth conditions.

**Figure 6:**
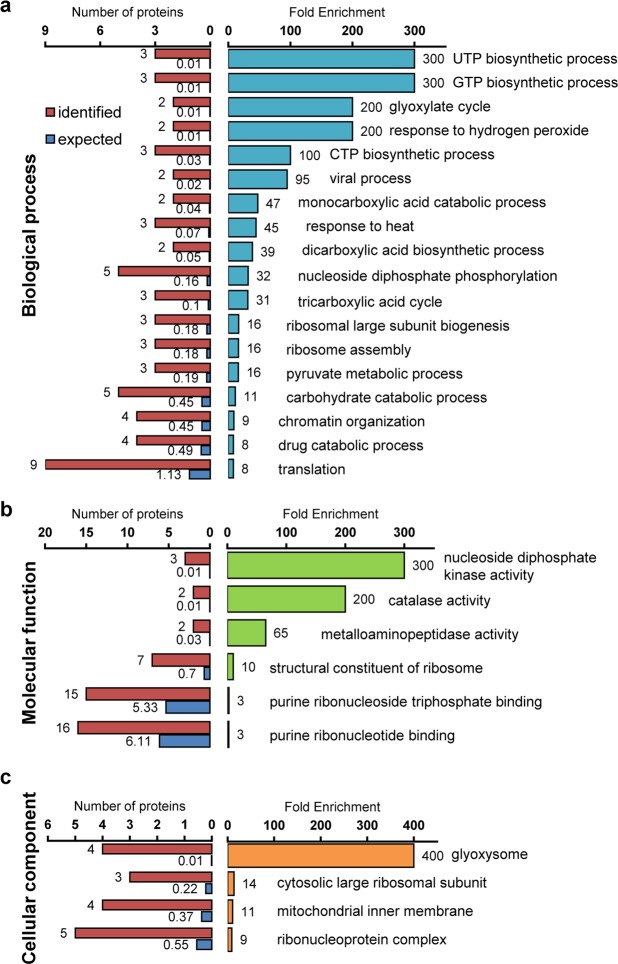
Effects of autophagy disruption on the biological process, molecular function and cellular component of endosperm cells in rice. GO enrichment analysis using the PANTHER Classification System permitted them to fall into 198 GO terms including biological process (**a**), molecular function (**b**), and cellular component (**c**). Proteins were categorized using the PANTHER databases. The top hierarchies are shown in each process. The significance of each GO term is calculated by comparing GO terms of input proteins (identified; red) with those in the whole transcriptome (expected; blue).

To analyze the effects of autophagy disruption in rice seeds, gene ontology (GO) enrichment analysis using the PANTHER Classification System was performed. The annotation of protein UniProt IDs using the PANTHER website permitted them to fall into 198 GO terms (FDR *P* < 0.05) including biological process, molecular function, and cellular component (Fig. 6, display only the highest hierarchy, and Supplementary Table 3). Eighty-five of all 145 proteins differentially expressed between WT and the *Osatg7-1* grains were mapped to 44321 reference genes contained in the whole rice genome, while the others (60 proteins) were unmapped. Among them, in the biological process group, response to hydrogen peroxide and response to heat were ranked at the top of the category (Fig. 6a). Moreover, in the molecular function and cellular component groups, both catalase activity and peroxisome-enzymatic pathways were ranked at the top of the category occupancy (Fig. 6b,c).

**Figure 7:**
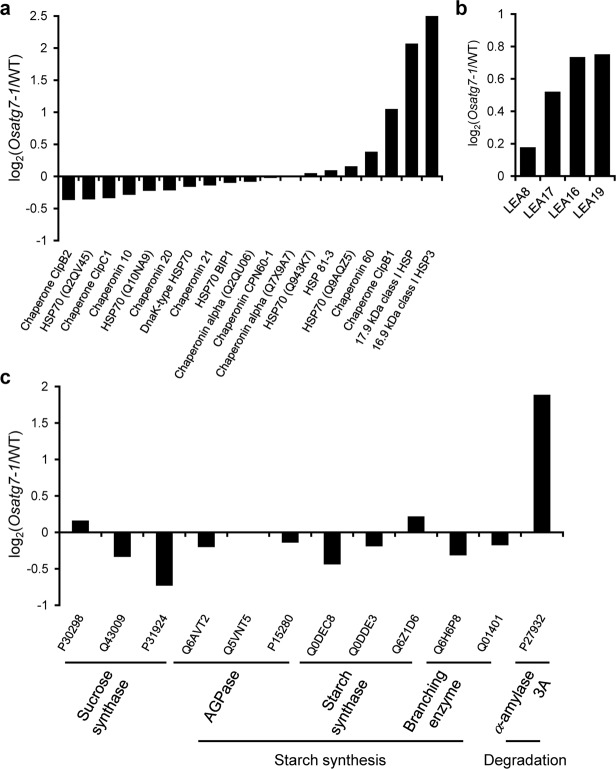
Proteomic comparisons of starch metabolism-related enzymes and stress-related proteins in chalky grains of WT and the *Osatg7-1* mutant. The comparative expression of heat shock-related proteins (**a**), LEA proteins (**b**) and starch metabolism-related enzymes (**c**) between WT and *Osatg7-1* mutant grains.

HSPs stabilize protein conformation, prevent aggregation, and, thus, maintain non-native proteins in a competent state for subsequent refolding as molecular chaperones in plants under heat stress^35–37^. Significant up-regulation of HSPs is a key part of the heat shock response^38,39^, and the levels of a variety of HSPs as well as chaperones were strongly up-regulated in the chalky grains of the *Osatg7-1* mutant (Fig. 7a). In addition, LEA proteins, which are involved in desiccation resistance as well as direct protection of other proteins or membranes, or renaturation of unfolded proteins^40,41^, highly accumulated in the chalky grains of the *Osatg7-1* mutant (Fig. 7b), suggesting that autophagy may be involved in protecting endosperm development from cellular damage caused by environmental stresses, such as heat or water loss, during rice seed maturation.

Autophagy is induced by abiotic stresses^42^, and autophagy-defective mutants are hypersensitive to environmental stresses in several plant species^43–47^. It is partly because autophagy plays a critical role in the degradation of potentially toxic misfolded/denatured proteins and their aggregates as a protein quality control mechanism^48–50^. The present results showed that grains of *Osatg7-1* mutant show whity chalky appearance under normal growth conditions, and the SEM analysis revealed smaller and sparser starch granules in the lateral region of the *Osatg7-1* endosperm than WT (Figs. 1 and 2). In rice, chalky zone sometimes occurs at the lateral region of the endosperm in developing kernels under high-temperature conditions^51^, which are called white-back grains. Ring-shaped chalkiness is suggested to be a cell-specific event associated with the disruption of starch accumulation^52^. In contrast, increase in the frequency of pitting in starch granules observed in the grains ripened under high temperature has been suggested to be due to the premature autolysis of starch by abnormal induction of *α*-amylases in rice^53,54^, wheat and maize^55^, implying that autophagy deficiency may lead to a similar phenotype as heat stress-induced starch autolysis during seed maturation in rice.

However, no significant differences have been observed in the contents and structure of amylose and amylopectin in starchy endosperms between the *Osatg7-1* mutant and WT grains (Supplementary Table 1). Slight alternation of amylopectin chain length has been shown to occur in the heat-induced chalky grains and long-length chains seem to increase in chalky grains^56^, suggesting some difference in the loosely-packed starch granules of the *Osatg7-1* mutant and the heat-stressed wild type plants.

In the proteome analysis followed by GO analysis, proteins associated with hydrogen peroxide were ranked at the top of the category occupancy (Fig. 6a). Moreover, the level of catalase, one of the major scavenging enzymes of reactive oxygen species (ROS), was also upregulated in the chalky grains of the *Osatg7-1* mutant (Fig. 6b). Autophagy-deficient mutants of Arabidopsis such as *atg5* are reported to over-accumulate ROS in leaves^18^. Autophagy is involved in the turnover of peroxisomes, which play a role in the degradation of hydrogen peroxide^57^. Moreover, autophagy is involved in the turnover of mitochondria as well as chloroplasts^58^, which induce over-accumulate ROS in environmental stress conditions, suggesting that the ROS scavenging system may be activated in the anthers of the *Osatg7-1* mutant to alleviate such oxidative damages. The relationship among autophagy, redox homeostasis and grain quality (enhanced frequency of chalky grains) as well as environmental stress adaptation would also be an important topic for future research.

### Involvement of autophagy in the regulation of starch turnover and *α*-amylases during rice seed maturation

The present omics analyses revealed that the disruption of autophagy clearly resulted in the differential expression of proteins related to a variety of metabolite and suggested that starch metabolic pathways are strongly affected (Fig. 5). Among them, *α*-amylase 3 A was more strongly up-regulated in the chalky grains of the *Osatg7-1* mutant than in WT grains (Fig. 7c). This result was consistent with those of the immunoblot analysis (Fig. 4b). In contrast, the expression levels of granule-bound starch synthases (GBSS), starch branching enzyme (SBE) BEIIb, ADP-glucose pyrophosphorylases (AGPases), and sucrose synthases (SuSy) were slightly lower in the *Osatg7-1* mutant than in WT (Fig. 7c). These results suggest that autophagy participates in the regulation of the starch degradation pathways of the rice endosperm during seed maturation, and the impaired balance between starch biosynthesis and degradation at the seed maturation stage may cause the imperfect filling of endosperm cells with starch granules in the *Osatg7-1* mutant.

As osmolytes and signaling molecules, soluble sugars participate in the response and adaptation of plants to environmental stresses^59^. Under salinity stress, photosynthetic carbon assimilation is inhibited, and starch turnover as well as an accumulation of soluble sugars are induced in rice leaves^60^. The production of soluble sugars, such as fructose, glucose, maltose, and sucrose, appeared to be at the expense of starch through the overexpression of *α*-amylase genes^31^. The accumulation of maltose may be due to starch degradation by the activation of *α*-amylase, which is a typical endoglucanase and produces oligosaccharides. High temperature stress promotes accumulation of glucose and sucrose in the mature grains of rice^24,61,62^. Moreover, in the developing seeds of rice, expression of the *α*-amylase genes *Amy1A*, *Amy1C*, *Amy3D*, and *Amy3E* were induced under high-temperature stress, and its suppression through an RNA interference (*RNAi*) strategy mitigated grain damage such as chalkiness^8^. In the present study, we found significant increase in the content of maltose as well as the activation of *α*-amylase isoforms in *Osatg7-1* grains grown under normal growth conditions (Figs. 3, 4 and 7). These results support *de novo* starch degradation by the abnormal activation of *α*-amylase in the chalky grains of the *Osatg7-1* mutant and suggest that its chalky-grain phenotype may be at least in part caused by enhanced sensitivity to environmental stresses.

### Concluding remarks

Overall, the present study revealed that autophagy plays essential roles in the regulation of starch and sugar metabolism as well as quality control of endosperm cells during seed maturation in rice. These findings shed light on a possible novel strategy for protection of grain quality and crop yield affected by environmental stresses. The relationship among autophagy induction throughout seed development and maturation, quality control in organelles and stress adaptation as well as metabolite regulation during seed maturation, is an important topic for future research.

## Methods

### Plant materials and growth conditions

Surface-sterilized seeds of wild-type rice *Oryza sativa* L. cv. *Nipponbare* (NB) and the *Osatg7-1* mutant^21^ were germinated on MS medium containing 0.8% agar and grown for 10 days in a growth chamber under long-day conditions (16-h light/8-h darkness, 28 °C). Seedlings were transplanted into soil and grown in the greenhouse at NIES in Tsukuba (Japan) or the paddy field at NIG in Mishima (Japan).

*Tos17*-insertional rice *Osatg7-1* mutant (*OsATG7*−/−), wild-type (*OsATG7* +/+), and heterozygous (*OsATG7* +/−) plants were selected in seed pools obtained from heterozygous plants by genomic PCR using the following primer mixture: *OsATG7* forward primer 5′-CATACTACCACCTCAGCTTGCTAG-3′, *Tos-17* forward primer 5′-ACTATTGTTAGGTTGCAAGTTAGTTAAGA-3′, and *OsATG7* reverse primer 5′-GCATTCAGGAAAACCTCGTATCG-3′. The original parental cultivars of the *Tos17*-insertional mutant and NB were also used as control plants for *Osatg7-1*.

### Scanning electron microscopic (SEM) and Electron Probe Micro Analysis (EPMA)

SEM and EPMA analyses of the mature rice endosperm was carried out as described previously^24^. Rice grains were cracked with a razor blade, and the cracked surfaces were coated with gold for 90 s using a vaporizer (IB-3, EIKO, Tokyo, Japan) and subjected to SEM (JSM6510LA, JEOL, Tokyo, Japan) or EPMA (EPMA-8705, Shimazu, Kyoto, Japan). Observation conditions were as follows: acceleration voltage, 10 kV; magnification, 1,000–10,000. These analyses were carried out using three biological replicates with at least four technical repetitions of rice grains per replication of the mounted specimens.

### Transmission electron microscopic (TEM) analysis

TEM analysis of the mature rice endosperm was carried out as described previously^63^. Samples were fixed with 4% w/v paraformaldehyde in Dullbecco’s PBS (−) (Nacalai Tesque, Kyoto, Japan) for 3 h. Specimens were rinsed in the same buffer and the samples were infiltrated overnight with D-PBS (−). Dehydrated specimens were embedded in LR White resin (London Resin, Hampshire, UK). Resin sections (200 nm) were cut with a diamond knife using Ultracut UCT. Ultrathin sections were stained with 2% w/v uranyl acetate. After staining, samples were examined with TEM (JEM-1220, JEM, Tokyo) at 80 kV.

### Measurement of soluble sugars and starch content

Rice grains were pulverized using a Shake Master NEO (Biomedical Science Inc.) with a stainless steel bead (6 mm, Biomedical Science Inc.) and three zirconia beads (3 mm, Nikkato Inc.) for 5 min at 1,750 rpm. Soluble sugars were extracted from 5–10 mg powdered rice grain with 1 mL of 80% EtOH containing 20 μg/mL methyl glucose (mGlc) as internal control for 1 h and the supernatant was recovered as soluble sugar fraction. The supernatant was completely evaporated by centrifugal dryer and then dissolved with ultrapure water. Contaminated protein in dissolved solution was removed with chloroform twice and then aqueous layer was recovered, evaporated, and then dissolved with ultrapure water again. The glucose, fructose, and maltose in dissolved solution were derivatized with *p-*aminobenzyl ethyl ester (ABEE) and those contents were determined with ABEE-UPLC system as described previously^64^. Sucrose was determined by the enzymatic method with invertase. Glucose released from sucrose in invertase solution containing 2 unit/mL invertase solution (Wako, Osaka, Japan) and 25 mM citrate at pH4.8 was determined as sucrose content by Glucose test Kit II (Wako, Osaka, Japan). Those detected sugar amounts were normalized with recovery rate of mGlc.

Starch content was determined by slightly modified method of “Resistant Starch Assay” (Megazyme). Insoluble residue above mentioned was rinsed with 80% EtOH again and then dried overnight at 65 °C. Crushed rice grain (2–3 mg) was hydrophilized with 20 μL of 100% EtOH and gelatinized in 180 μL of maleate buffer (pH4.8) for 10 min at 65 °C. Starch in gelatinized suspension was digested in amylase solution containing 100 unit/mL *α*-amylase (Megazyme) and 5.5 unit/mL amyloglucosidase (Megazyme) in maleate buffer (pH4.8) for 20 h at 37 °C with shaking. The liberated glucose from insoluble residue was determined as starch content by Glucose Test Kit II (Wako, Osaka, Japan).

### Preparation of starch granules and amylopectin for gel filtration

The method used to assess the composition of carbohydrate content in endosperm was performed as described previously^34^. The eluate refractive index was measured with an RI-8020 detector (Tosoh, Japan). Fractions I, II, and III were divided at the minimum values of the refractive index curves. The contents of apparent amylose, true amylose, and extra-long chains in starch were calculated from the ratios of fractions I, II, and III. The contents of each fraction were confirmed by mixing the eluate with iodine and measuring the λ_max_ value with the spectrophotometer as described previously^65^.

### Measurement of *α*-amylase activity in embryonic rice

*α*-Amylase activity was assayed by a modification of the method described previously^66^. Mature rice grains were ground in liquid nitrogen to fine powder and then suspended in a 10-fold volume of extraction buffer (20 mM Tris-HCl, pH 7.5, 0.01% (w/v) Triton X-100) at 4 °C for 10 min. The suspensions were centrifuged at 15,000 × *g* at 4 °C for 15 min, and the supernatant was used to determine *α*-amylase activity. One-hundred μL of substrate solution (50 mM acetate buffer, pH 5.3, 0.5 mM CaCl_2_, 5 mM EDTA, pH7.5 and 0.34% soluble potato starch) and 100 μL of the supernatant was incubated at 37 °C for 5 min and then the reaction was stopped by the addition of 250 μL KI solution (1 mM). After dilution of with D.W. (up to 1 mL), *A*_620_ was determined to measure the *α*-amylase activity. The activity of *α*-amylase was calculated using the following equation: units of *α*-amylase activity = [10 − (*A*_620_ Sample/ *A*_620_ Blank) × 10]. The data were corrected by the protein content of the respective extracts determined by the Pierce 660 nm Protein Assay Kit (Thermo Fisher Scientific) using bovine serum albumin (BSA) as a standard.

### Immunoblot analysis

The immunoblot analysis was performed as described previously^24^. Crude protein samples from mature grains of rice were separated on a 12% SDS-PAGE gel and blotted onto a PVDF membrane. The membrane was blocked in PBST buffer (8.1 mM Na_2_HPO_4_, 137 mM NaCl, 1.5 mM KH_2_PO_4_, 2.7 mM KCl and 0.1% Tween-20, pH7.5) with 5% fat-free milk 25 °C for 1 h. Blots were incubated with specific antibodies detecting the amylase isoforms^30^ at 25 °C for 1 h, and then with horseradish peroxidase (HRP)-linked anti-rabbit immunoglobulin G. Bands were quantitatively detected using chemiluminescent HRP substrate ECL Prime (Amersham) and the chemiluminescent analyzer, LAS3000 (GE Healthcare). Signals were normalized by the intensity of Coomassie brilliant blue staining with the corresponding protein.

### Proteomic analysis

Two hundred mg of starchy endosperm prepared from the opaque part of chalky grains or the corresponding part of perfect grains were ground in liquid nitrogen to fine powder and then suspended in 7 M urea, 2 M thiourea, 3% (w/v) CHAPS, 1% (v/v) Triton X-100, and 10 mM DTT. The suspensions were centrifuged at 10,000 × *g* at 4 °C for 5 min. The supernatants were mixed with 1/10 volume of 100% (w/v) trichloroacetic acid, incubated on ice for 15 min, and centrifuged at 10,000 × *g* at 4 °C for 15 min. The resulting protein precipitates were washed 3 times in ice-cold acetone and resuspended in 8 M urea. Protein concentration was determined by the Pierce 660 nm Protein Assay Kit (Thermo Fisher Scientific).

The procedure of quantitative shotgun proteomic analysis was essentially identical as described previously^24^. Each protein preparation (50 μg) was digested in 20 μL of endoproteinase Lys-C (1 μg/ μL) at 37 °C for 3 h, then diluted to 10 times volume by ultrapure water (18.2 MΩ cm). The diluted samples were further digested in 200 μL of trypsin (1 μg/ μL) at 37 °C for 12 h. iTRAQ labeling of peptides were carried out with 4-plex iTRAQ tags the manufacturer’s protocol (Sciex), and the resultant 4 iTRAQ-labeled peptide samples were mixed. iTRAQ analysis was performed by using a DiNa-A-LTQOrbitrap-XL system. The iTRAQ labeled peptides were loaded on a trap column (HiQ sil C-18 W-3; 0.5 mm i.d. × 1 mm, 3 μm particle size) with buffer A consisting of 0.1% (v/v) formic acid and 2% (v/v) acetonitrile in water using a DiNa-A system (KYA Tech., Tokyo, Japan). A linear gradient from 0 to 33% buffer B (0.1% formic acid and 80% acetonitrile in water) for 600 min, 33 to 100% B for 10 min and back to 0% B in 15 min was applied, and peptides eluted from the HiQ sil C-18 W-3 column were directly loaded on a separation column (MonoCap C18 High Resolution 2000; 0.1 mm i.d. × 2000 mm, 2 μm pore size). The separated peptides were introduced into an LTQ-Orbitrap XL mass spectrometer (Thermo Fisher Scientific) with a flow rate of 300 nL min−1 and an ionization voltage 1.7–2.5 kV.

Liquid chromatography-MS/MS (LC-MS/MS) spectrometer was operated using Xcalibur 2.0 software (Thermo Fisher Scientific). The mass range selected for MS scan was set to 350–1,600 m/z and the top three peaks were subjected to MS/MS analysis. Full MS scan was detected in the Orbitrap, and the MS/MS scans were detected in the linear ion trap and Orbitrap. The normalized collision energy for MS/MS was set to 35 eV for collision-induced dissociation (CID) and 45 eV for higher-energy C-trap dissociation (HCD). High resolution of Fourier transform mass spectrometer (FTMS) was maintained at 60,000 resolutions. Divalent or trivalent ions were subjected to MS/MS analysis in dynamic exclusion mode, and proteins were identified with Proteome Discoverer v. 1.4 software and the SEQUEST HT (Thermo Fisher Scientific) and Ms Amanda^67^ search tool using the UniProt (http://www.uniprot.org/) *O. sativa* subsp. japonica database (63,535 proteins) with the following parameters: enzyme, trypsin; maximum missed cleavages site, 2; peptide charge, 2 + or 3 + ; MS tolerance, 5 ppm; MS/MS tolerance, ± 0.5 Da; dynamic modification, carboxymethylation (C), oxidation (H, M, W), iTRAQ 4-plex (K, Y, N-terminus). False discovery rates were < 1%.

### Gene ontology analysis

Gene Ontology (GO) enrichment analyses of GO terms were conducted on each set of differentially expressed proteins to the reference list including all genes in *Oryza sativa* genome (released 2018–11–15) by using the PANTHER overrepresentation test. Enriched GO terms were calculated false discovery rate (FDR) settings and statistically analyzed by Fisher’s exact test (*P* < 0.05).

### Statistical analysis

Significance was assessed using an unpaired Student’s *t*-test at *P* < 0.05.

### Accession codes

Sequence data from this article can be found in the GenBank databases under the following accession numbers: *OsATG7* (Os01g0614900), *AmyI-1* (Os02g0765600), *Amy3A* (Os09g0457400), *AmyII-3* (Os08g0473600), *AmyII-4* (Os08g0473900), *AmyII-5* (Os09g0457600), *AmyII-6* (Os09g0457800), and *ONG2* (Os06g0675700).

## Supplementary information

Supplementary Figures

Table S1

Table S2

Table S3
