# Supplementary material for: Essential roles of autophagy in metabolic regulation in endosperm development during rice seed maturation
Source: Sci Rep. 2019 Dec 6;9:18544. doi: 10.1038/s41598-019-54361-1 (PMC6898296; doi:10.1038/s41598-019-54361-1)
Supplement: Supplementary file 1 — Supplementary Figures [file 41598_2019_54361_MOESM1_ESM.pdf]

Essential roles of autophagy in metabolic regulation in endosperm development during rice seed maturation

Yuri Sera[1,12], Shigeru Hanamata[1,2,9,12], Shingo Sakamoto[3], Seijiro Ono[4,11], Kentaro Kaneko[2], Yuudai Mitsui[2], Tomoko Koyano[1], Naoko Fujita[5], Ai Sasou[6,10], Takehiro Masumura[6], Hikaru Saji[7], Ken-Ichi Nonomura[4], Nobutaka Mitsuda[3], Toshiaki Mitsui[2], Takamitsu Kurusu[1,8,9]*, Kazuyuki Kuchitsu[1,9]*

[1]Department of Applied Biological Science, Tokyo University of Science, 2641 Yamazaki, Noda, Chiba, 278-8510, Japan, [2]Graduate School of Science and Technology, Niigata University, 2-8050 Ikarashi, Niigata, 950-2181, Japan, [3]Bioproduction Research Institute, National Institute of Advanced Industrial Science and Technology (AIST), Tsukuba, Ibaraki, 305-8566, Japan, [4]Plant Cytogenetics Laboratory, National Institute of Genetics, 1111 Yata, Mishima, Shizuoka, 411-8540, Japan, [5]Department of Biological Production, Akita Prefectural University, 241-438 Shimoshinjo Nakano Kaidobata-Nishi, Akita City, Akita, 010-0195, Japan, [6]Laboratory of Genetic Engineering, Graduate School of Life and Environmental Sciences, Kyoto Prefectural University, Shimogamo, Kyoto, 606-8522, Japan, [7]Center for Environmental Biology and Ecosystem Studies, National Institute for Environmental Studies (NIES), Onogawa, Tsukuba, Ibaraki, 305-8506, Japan, [8]Department of Mechanical and Electrical Engineering, Suwa University of Science, 5000-1 Toyohira, Chino, Nagano, 391-0292, Japan, [9]Imaging Frontier Center, Tokyo University of Science, 2641 Yamazaki, Noda, Chiba 278-8510, Japan, [10]Division of Mucosal Immunology, Institute of Medical Science, The University of Tokyo, 4-6-1 Shirokanedai, Minato-ku, Tokyo, 108-8639 Japan, [11]Department of Developmental Biology, Institute for Plant Sciences and Microbiology, University of Hamburg, 22609 Hamburg, Germany, [12]These authors contributed equally to this work: Yuri Sera and Shigeru Hanamata.

*Co-corresponding authors:

Kazuyuki Kuchitsu, Department of Applied Biological Science, Tokyo University of Science, 2641 Yamazaki, Noda, Chiba, 278-8510, Japan, E-mail: kuchitsu@rs.noda.tus.ac.jp, Tel.: +81-4-7122-9404, Fax: +81-4-7123-9767

Takamitsu Kurusu, Department of Mechanical and Electrical Engineering, Suwa University of Science, 5000-1 Toyohira, Chino, Nagano, 391-0292, Japan, e-mail: kurusu@rs.sus.ac.jp, Tel.: +81-266-73-9826

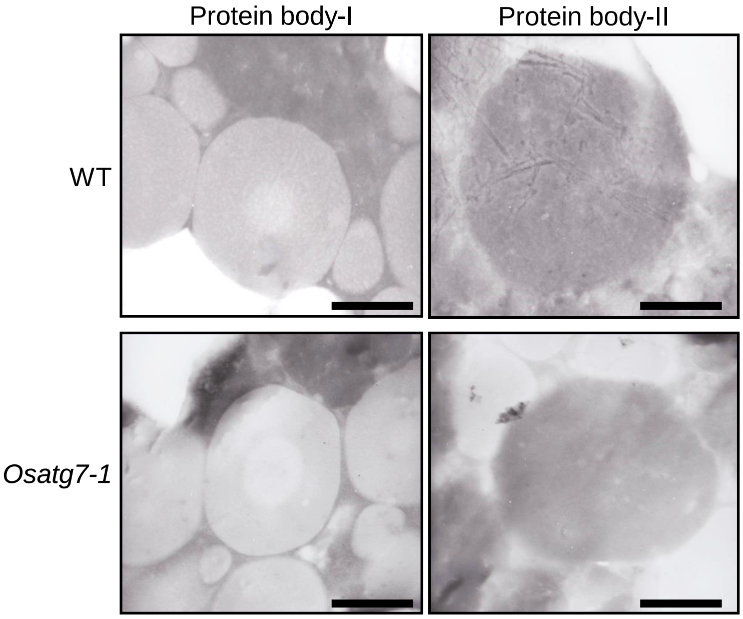

Figure S1. Effects of autophagy disruption on protein body structure in the endosperm of rice. Ultrastructure of protein body (PB)-I and II in the starch endosperm of WT (Upper) and the *Osatg7-1* mutant (Lower) at the seed developing stage. Scale bar: 1 mm. Similar results were obtained from 5 independent samples. No significant differences were observed in the structures of protein bodies between WT and the *Osatg7-1* mutant.

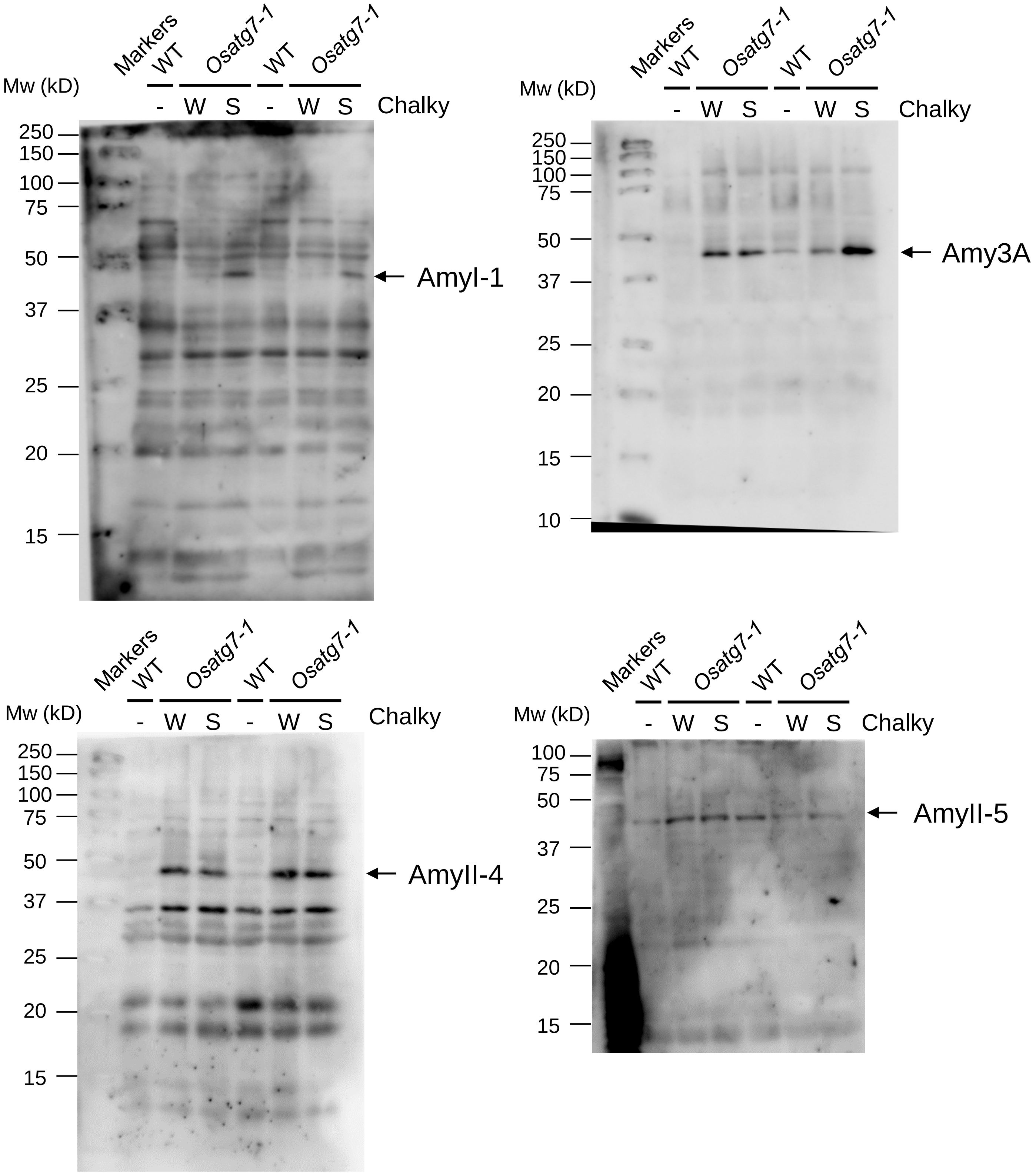

Figure S2. The full-length blots shown in Figures 4B.

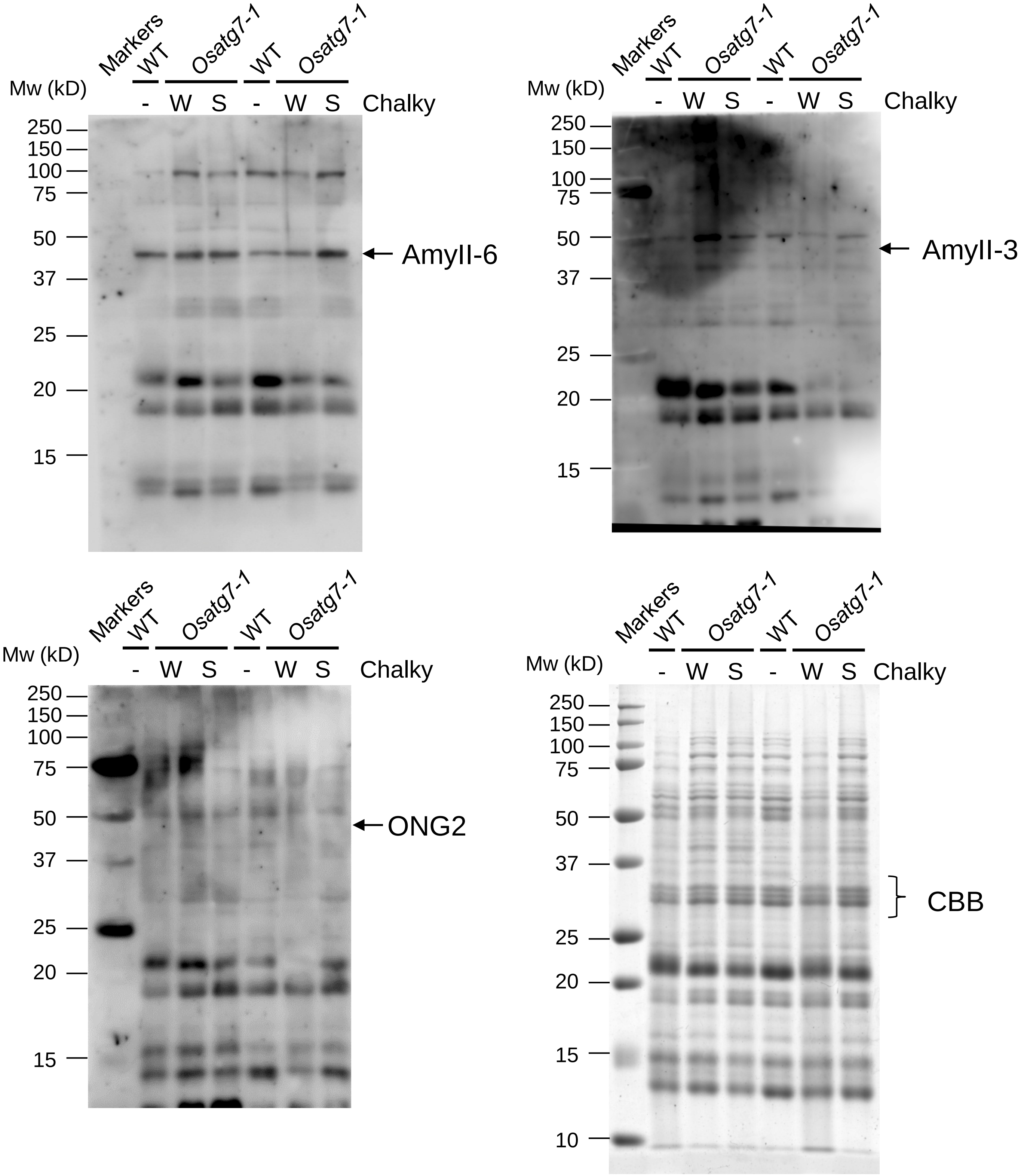

Figure S3. The full-length blots shown in Figures 4B.
